# Corneal Endothelial Characteristics, Central Corneal Thickness, and Intraocular Pressure in a Population of Chinese Age-Related Cataract Patients

**DOI:** 10.1155/2017/9154626

**Published:** 2017-05-25

**Authors:** Yingmei Li, Zhentao Fu, Jian Liu, Miao Li, Yuguang Zhang, Xinyi Wu

**Affiliations:** ^1^Department of Ophthalmology, The Second People's Hospital of Jinan, No. 148 Jingyi Road, Jinan, Shandong 250001, China; ^2^Academy of Preventive Medicine, Shandong University, Jinan, China; ^3^The Department for Chronic and Noncommunicable Disease Control and Prevention, Shandong Center for Disease Control and Prevention, Jinan, Shandong, China; ^4^Department of Ophthalmology, Qilu Hospital Affiliated Shandong University, Jinan, Shandong 250012, China

## Abstract

**Purpose:**

To describe corneal endothelial characteristics, central corneal thickness, and intraocular pressure in a population of Chinese age-related cataract patients and to determine the effects of age, gender, hypertension, and body mass index (BMI).

**Methods:**

1551 eyes were examined preoperatively. The parameters measured were endothelial cell density (CD), average cell area (CA), coefficient of variation (CV), cell hexagonality (HEX), central corneal thickness (CCT), intraocular pressure (IOP), and axial length (AL).

**Results:**

There were significant differences in CV and BMI between genders. There was a significant decrease in CD, CCT, and IOP and, conversely, an increase in CA and BMI with increasing age. The patients who suffered from hypertension had bigger CA, less CD, and lower HEX than the patients who did not suffer from hypertension. CD has a negative correlation with age and CV and a positive correlation with CCT, CCT has a positive association with CD and IOP, and IOP had a negative relationship with age and a positive relationship with CCT, CA, and HEX.

**Conclusions:**

Normative data for the corneal endothelium, central corneal thickness, and intraocular pressure in the normal age-related cataract patients are reported which will serve as a baseline for comparative studies about cataract.

## 1. Introduction

Age-related cataract remains the most common cause of blindness throughout the world [1, 2]. At present, cataract extraction is the only treatment option available which is the most frequently performed in the world. It is well established that cataract extraction decreases the number of corneal endothelial cells, which can lead to corneal edema, even bullous keratopathy when endothelial cell density significantly decreases. The degree to which the endothelial cell is affected depends on many factors, such as surgical technique, phacoemulsification energy, use of ophthalmic viscoelastic devices [3–5], and preexisting corneal endothelial cell characteristics. Because the human corneal endothelium is a single layer of predominantly six-sided cells with a limited regenerative capacity in vivo [6–8] which can stop aqueous humor into the extracellular space and it is crucial for maintaining corneal clarity, it is significant to know the corneal endothelial cell status of cataract patients before surgery. In this study, we described the preoperative central corneal endothelium cells as a baseline study for a clinical trial on age-related cataract surgery.

Many factors have been documented to affect the morphologic features of corneal endothelial cells such as age [9–11], gender [12–15], different ethnic groups [12–16], central corneal thickness [17], axial length [18–22], diabetes mellitus [23–25], ocular trauma [26], intraocular surgery, and various diseases.

This prospective study reported corneal endothelial characteristics, intraocular pressure, and central corneal thickness in normal age-related cataract patients and defined the impacts of gender, age, hypertension, and BMI on these parameters.

## 2. Materials and Methods

This study was a cross-sectional study that was conducted between January 2016 and December 2016. 1551 eyes of 1269 age-related cataract patients between 40 and 96 years of age were examined preoperatively with noncontact specular microscopy at The Second People's Hospital of Jinan in Shandong province, China. The 1269 patients were of the same Chinese ethnic origin, the Han Nationality, and were the inhabitants of the Shandong region.

### 2.1. Selection Criteria

The patients were normal age-related cataract patients who were in good general health and had a negative history of intraocular surgery, corneal surgery, ocular trauma, increased intraocular pressure, pseudoexfoliation syndrome, glaucoma, uveitis, keratopathy, myopia, diabetes mellitus, contact lens wear, and other diseases that may have caused cataract. Because the opacity of the lens could affect the results of eye refraction, we selected patients by reference to the axial length of the eye. The patients whose axial length of the eye was greater than 25 mm were excluded from the study. Hypertension patients were included in the study whose blood pressure was controlled and stable. Our study was performed in accordance with the tenets of the Declaration of Helsinki and informed consent was obtained from all patients.

### 2.2. Examinations

Routine examinations included height, weight, slit-lamp examination, intraocular pressure, and axial length. Systolic blood pressure (SBP) and diastolic blood pressure (DBP) were measured with a mercury sphygmomanometer on the left arm between 9:00 am and 11:00 am. Height and weight were measured by using a height and weight scale, and body mass index (BMI) was calculated as weight/height^2^. Axial length was measured with A-scan biometry. IOP was measured three times with a noncontact tonometer (Canon TX-F, Canon, Tokyo, Japan) between 9:00 am and 11:00 am, and the mean value was used for analysis. All the patients underwent an examination with a noncontact specular microscope (Topcon, SP3000P, Tokyo, Japan) to evaluate the endothelial cell density (CD), average cell area (CA), coefficient of variation (CV) in cell area, cell hexagonality (HEX), and central corneal thickness (CCT). These parameters provided an index of the functional status of corneal endothelial layer.

The SP3000P is a noncontact optical instrument and one of the most popular instruments used in clinical practice—simultaneously providing pachymetric measurements and specular microscopy. In this study, the measurements were obtained in the semiautomatic mode of the instrument and three examiners who were trained by the same instructor performed all measurements. The patient's head was positioned on the chin rest, and the patient was asked to look straight ahead into the built-in fixation target. Three images from the central cornea were taken, and the best image was saved for analysis. The examiner hand-digitized the center of each cell in a contiguous group of 50 cells at least. CD, CA, CV, HEX, and CCT were automatically evaluated and calculated by a computer program incorporated into the instrument.

### 2.3. Data Handling and Statistical Analysis

Linear regression analysis was used to determine the changes in endothelial cell parameters, CCT, and IOP with age, and an unpaired *t-*test was used to compare all these parameters between sexes. Multivariate linear regression was performed to explore the association between CD, IOP, CCT (dependent factor), and other parameters (independent factors), respectively. All statistical analyses were performed using SPSS 18.0 statistical analysis software, and *P* values less than 0.05 were considered as statistically significant.

## 3. Results

A total of 1551 eyes of 1269 patients were enrolled in the study. Mean values for the parameters measured are shown by gender in Table 1. The mean age, mean CCT, mean IOP, and mean AL were 69.6 ± 11 years (range 40–96 years), 515.1 ± 33 *μ*m (range 467–611 *μ*m), 14.5 ± 3 mmHg (range 6.5–21 mmHg), and 23.2 ± 1.7 mm (range 19.9–25 mm), respectively. The mean CD, mean CA, mean CV, and mean HEX were 2454 ± 473.2 cells/mm^2^ (range 899–4016 cells/mm^2^), 427.5 ± 107.4 *μ*m^2^ (range 371–666 *μ*m^2^), 36.3 ± 13.4% (range 20–59%), and 52 ± 26% (range 18–100%), respectively. The mean BMI, mean SBP, and mean DBP were 23 ± 2.8 kg/m^2^ (range 20–24 kg/m^2^), 127.2 ± 16.2 mmHg (range 90–155 mmHg), and 81.2 ± 10.1 mmHg (range 60–100 mmHg), respectively. There were no statistically significant differences in age, CD, CA, HEX, CCT, IOP, SBP, DBP, and AL between genders (all *P* values > 0.05). However, there were significant differences in CV and BMI between genders (both *P* values < 0.05). Men had a CV and a BMI that were higher than those of women (CV: female 35.3 ± 12.5%; male 36.9 ± 15%) (BMI: female 22.8 ± 2.7 kg/m^2^; male 23.2 ± 2.9 kg/m^2^). There were 6 eyes (0.39%) with CD below 1000 cells/mm^2^, 26 eyes (1.68%) with CD below 1500 cells/mm^2^, 182 eyes (11.73%) with CD below 2000 cells/mm^2^, and 196 eyes (12.64%) with CD above 3000 cells/mm^2^. For most of the eyes (75.63% of the 1551 eyes), the CD was between 2000 and 3000 cells/mm^2^.

The endothelial cell parameters, CCT, IOP, and AL, in different age groups are shown in Table 2. Regression analysis showed that with the increase in age there was a statistically significant decrease in CD (*R* = −0.173, *P* < 0.0001), CCT (*R* = −0.086, *P* < 0.001), and IOP (*R* = −0.127, *P* < 0.0001) and conversely an increase in CA (*R* = 0.147, *P* < 0.0001) and BMI (*R* = 0.137, *P* < 0.001). There was a gradual increase in the rate of cell loss with advancing age, except for the seventh and eighth decades of life. The highest rate of cell loss was noted in the last decade of life (≥90 years old) in the studied population (1.69%). In addition, the change in AL, CV, and HEX were not found to be dependent on age (all *P* values > 0.05).

In Table 3, the subjects were classified into two groups according to whether or not they have suffered from hypertension. There were statistical differences in age, CD, CA, HEX, and BMI between two groups (all *P* values < 0.05). The patients who suffered from hypertension were much older, had bigger CA and BMI, less CD, and lower HEX than the patients not suffering from hypertension. There were no statistical differences in CCT, IOP, CV, and AL between two groups (all *P* values > 0.05).

The relationship among age, sexes, HBP, AL, CCT, CD, CA, CV, HEX, and BMI was studied using multiple-factor regression which was performed using CD, CCT, and IOP as dependent factor, respectively, and other parameters as independent factors. The results revealed that CD has a negative correlation with age (*P* = 0.001), CA (*P* < 0.001), and CV (*P* = 0.003); a positive correlation with CCT (*P* = 0.044); and no correlation with other parameters (*P* > 0.05) (Table 4). IOP had a negative relationship with age (*P* < 0.001) and AL (*P* = 0.011) and a positive relationship with CCT, CA, CV, and HEX (*P* < 0.05) (Table 5). CCT has a positive association with CD (*P* = 0.044) and IOP (*P* < 0.001) (Table 6).

## 4. Discussion

Many studies have been published to describe the relationship of endothelial cell density and morphology to age [9–15], gender [12–15], and ethnicity [12–16] in different populations. In this study, the subjects were normal age-related cataract patients who all were Han Nationality inhabitants of the Shandong region. Because corneal endothelial cell status could be affected by systemic and ocular conditions [18–20, 23–25], our subjects were in good general health and had a negative history of intraocular surgery, corneal surgery, ocular trauma, increased intraocular pressure, pseudoexfoliation syndrome, glaucoma, uveitis, keratopathy, high myopia, diabetes mellitus, contact lens wear, and other diseases that might cause cataract. The aim was to have a better understanding of corneal endothelial morphologic features in normal age-related cataract patients. Based on our sample size, we were confident that the analysis of cell data reflected real features in the population of the Shandong region.

It is well known that the density of the corneal endothelial cells was a pivotal factor in maintaining long-term corneal transparency. A normal cell density was crucial for maintaining normal function [6]. When the number of corneal endothelium fell below a critical number, it would cause bullous keratopathy. Some studies have described “at-risk” endothelial cell densities as being less than 2000 cells/mm^2^ [26, 27]. Some studies have showed the endothelial cell density of 1000 cells/mm^2^ as the threshold for acceptable risk [28]. In this study population, the mean CD was 2454 ± 473.2 cells/mm^2^. Most patients (75.63%) had normal CD values (2000~3000 cells/mm^2^), few patients (11.73%) had low CD values (<2000 cells/mm^2^), and very few patients (0.39%) had “threshold” CD values (<1000 cells/mm^2^) who might not tolerate cataract surgery because of the risk of corneal endothelial decompensation. In addition to the endothelial cell density, polymegethism (CV), and polygonality (HEX) were also important parameters of the corneal endothelium which had been used as the index of the extent of variation in cell area and in cell shape. A high HEX and low CV were also seen as an indicator of a healthy corneal endothelium. In this study, the mean CV and mean HEX were 36.3 ± 13.4% and 52 ± 26% which were consistent with other research [14, 29–31].

Previous studies have found that there was a continuous cell loss of 0.3%–0.5% per year roughly [32–34] and an increase in CA and CV [12, 13, 15] with increasing age. In this study, there was a statistically significant decrease in CD and an increase in CA with increasing age. CD had a negative correlation with age, CA, and CV. The rate of cell loss was 0.58% per year which was a little bit higher than that described in other studies. There was a higher loss of cells in the sixth and ninth decade of life, and the highest rate of loss was in the ninth decade. The variation in the cell loss rate in different age groups in our studies was not exactly the same to the results in other studies [12–16]. The reason for the difference may be related to our different studied populations and different age groupings. In this study, the subjects were aged from 40 to 96 years who were divided into six age groups and the '70s decade, '80s decade, and '90s decade were divided separately into three age groups. When those three groups were merged into one group, the rate of cell loss would de decreased, similar to that described in other studies. In addition, the change in AL, CV, HEX, SBP, and DBP were not found to be dependent on age in our studied population. The above results indicated that most normal age-related cataract patients had the number of corneal endothelial cells which was sufficient for cataract surgery. However, due to some unclear reasons, there were still a small number of normal patients whose corneal endothelial cells were reduced in different degrees. These demonstrated that the routine endothelial cell density examination preoperatively was very necessary for age-related cataract patients, especially for the patients over the age of 90 who had less endothelial cell density and harder crystal nucleus relatively.

The relationship between genders and endothelial cell characteristics was different in different studies. Some investigators reported differences between sexes [12–15], but others did not find any statistically significant differences between them [35–37]. In this study, 1551 subjects included 718 eyes from male and 833 eyes from female. There were no statistically significant differences in age, CD, CA, HEX, CCT, IOP, SBP, DBP, and AL between genders (all *P* values > 0.05), except for CV and BMI. Men had a CV and a BMI that were higher than those of women (Table 1).

There are many hypertensive patients in people with senile cataract. Our study included some hypertensive patients whose blood pressure was controlled and stable. Previous studies on the effect of blood pressure on corneal endothelium were few, and these studies showed that there were differences between the corneal endothelium in patients with hypertension and those without hypertension [38–40]. In this study, 890 eyes were from hypertensive patients and 661 eyes were from the patients without hypertension (Table 3). We compared these two groups and found that the hypertensive patients were much older, had bigger CA and BMI, less CD, and lower HEX than the patients without hypertension. Our results were consistent with the previous results. These results suggested that we should pay special attention to the protection of endothelial cells in cataract surgery for hypertensive patients.

Central corneal thickness and intraocular pressure are very important parameters for the eyes. Previous studies have examined the relationship between CCT, IOP, and age in different normal populations. The results showed a positive association between CCT and IOP [41–44], but found the distributions of CCT and IOP with age conflicting. Some results suggested that CCT is independent of the age [45–47], some results indicated that CCT decreases with age [48–50], some studies reported a positive correlation between increasing age and IOP [51–54], and some studies reported an inverse relationship of age and IOP [55, 56]. In this study, there was also a positive correlation between CCT and IOP (Tables 5 and 6). The regression analysis showed that with increase in age there was a significant decrease in both CCT and IOP (Table 2), while the multiple-factor regression analysis showed that IOP had a negative association with age but CCT had no correlation with age. Therefore, we could determine that the influence of age, CCT, and IOP is complex and cannot be examined in isolation.

Previous articles have confirmed that high intraocular pressure could lower the endothelial cell density [55–57], and when the amount of corneal endothelium was reduced to a certain extent, it could lead to corneal edema, increased corneal thickness, and even corneal decompensation. Many studies have been conducted to investigate a possible correlation between central corneal thickness and endothelial cells in various diseases [58–60], but little was known about the relationship between central corneal thickness, intraocular pressure, and endothelial cells in normal cataract eyes. In this study, multivariate regression analysis showed that CCT and CD were positively correlated (Tables 4 and 6) and IOP and CD were not significantly correlated (Tables 4 and 5). The results suggested that we should pay attention to the CCT value if the CD value was abnormal in the preoperative examination.

In conclusion, our values derived from a relatively large studied population established a normative database for endothelial parameters, CCT and IOP, in age-related cataract patients of Shandong province. For normal age-related cataract patients, age and CCT were both the main determinant in CD, while IOP, AL, BMI, and HBP seemed to have no effect on it. But IOP and HBP had effects on other corneal endothelial characteristics, such as CA, CV, and HEX. The results will aid clinical research in cataract surgery. Further clinical research of the same population should be undertaken to give the preoperative and postoperative changes of these parameters. Further research of other different populations with diseases such as glaucoma, uveitis, high myopia, and diabetes mellitus should be undertaken to give different results.

## Figures and Tables

**Table 1:** Characteristics of the subjects.

	Male (*n* = 718)	Female (*n* = 833)	*P*	Total (*n* = 1551)
	Mean ± SD	Mean ± SD		Mean ± SD
Age (yr)	69.2 ± 12.1	70.0 ± 9.9	0.15	69.6 ± 11
AL (mm)	23.22 ± 1.9	23.18 ± 1.5	0.19	23.2 ± 1.7
IOP (mmHg)	14.6 ± 2.9	14.5 ± 3	0.74	14.5 ± 3
CCT (*μ*m)	514.2 ± 30.9	516 ± 34.7	0.29	515.1 ± 33
CA (*μ*m^2^)	432.3 ± 118.2	423.4 ± 97	0.10	427.5 ± 107.4
CD (cells/mm^2^)	2458.2 ± 510.3	2450.5 ± 438.9	0.75	2454 ± 473.2
CV (%)	36.9 ± 15	35.3 ± 12.5	0.02	36.3 ± 13.4
HEX (%)	50.7 ± 26.2	53.2 ± 25.7	0.06	52 ± 26
SBP (mmHg)	127.8 ± 16.9	126.6 ± 15.6	0.15	127.2 ± 16.2
DBP (mmHg)	81.6 ± 10.5	80.9 ± 9.6	0.17	81.2 ± 10.1
BMI (kg/m^2^)	23.2 ± 2.9	22.8 ± 2.7	0.005	23 ± 2.8

AL: axial length; IOP: intraocular pressure; CCT: central corneal thickness; CA: average cell area; CD: endothelial cell density; CV: coefficient of variation in cell area; HEX: cell hexagonality; SBP: systolic blood pressure; DBP: diastolic blood pressure; BMI: body mass index.

**Table 2:** AL, IOP, CCT, CA, CD, CV, HEX, and BMI in different age groups.

Age group	40–49	50–59	60–69	70–79	80–89	≥90	Total	*P* value	*R*
Count	74	207	397	549	292	32	1551		
Age (yr)	43.99 ± 3.06	55.64 ± 2.9	64.56 ± 2.99	73.84 ± 2.89	82.49 ± 2.08	93.19 ± 1.57	69.64 ± 10.98		
AL (mm)	23.77 ± 2.07	23.31 ± 1.76	23.23 ± 1.65	23.03 ± 1.5	23.218 ± 1.68	23.38 ± 1.81	23.19 ± 1.7	0.07	−0.028
IOP (mmHg)	15.45 ± 2.79	14.59 ± 3.07	14.96 ± 2.66	14.42 ± 2.85	14.11 ± 3.3	14.1 ± 4.19	14.57 ± 2.97	<0.0001	−0.127
CCT (*μ*m)	517.09 ± 27.62	519.51 ± 40.51	515.74 ± 35.15	513.73 ± 31.32	517.42 ± 26.26	478.31 ± 22.73	515.13 ± 33.01	<0.001	−0.086
CA (*μ*m^2^)	365.84 ± 44.19	393.92 ± 72.29	453.05 ± 139.68	412.76 ± 71.64	450.72 ± 128.11	512.19 ± 47.4	427.52 ± 107.41	<0.0001	0.147
CD (cells/mm^2^)	2770.9 ± 319.8	2622.7 ± 478.2	2357.4 ± 507.6	2491.5 ± 372.2	2368.4 ± 543.8	1968.6 ± 178.8	2454.0 ± 473.2	<0.0001	−0.173
CV (%)	25.34 ± 7.54	31.24 ± 7.38	37.46 ± 15.46	39.17 ± 17.71	34.57 ± 10.38	29.81 ± 8.17	36.26 ± 13.44	0.063	0.025
HEX (%)	53.62 ± 16.97	53.84 ± 24.75	50.44 ± 25.68	51.93 ± 27.46	54.71 ± 25.81	34.56 ± 21.99	52.05 ± 25.95	0.137	−0.038
BMI (kg/m^2^)	22.52 ± 2.96	22.74 ± 2.78	23.08 ± 3.17	23.12 ± 2.58	23.19 ± 2.76	22.9 ± 2.69	23.03 ± 2.8	<0.001	0.137

**Table 3:** Comparison between the mean values of age, AL, IOP, CCT, CA, CD, CV, HEX, and BMI in the patients with hypertension (group 1) and the patients without hypertension (group 2).

	Group 1 (*n* = 890)	Group 2 (*n* = 661)	*P*
	Mean ± SD	Mean ± SD	
Age (yr)	72.74 ± 9.37	67.34 ± 11.52	0.000
AL (mm)	23.23 ± 0.94	23.16 ± 0.86	0.133
IOP (mmHg)	14.39 ± 3.11	14.64 ± 2.76	0.101
CCT (*μ*m)	515.92 ± 34.95	514.07 ± 30.17	0.276
CA (*μ*m^2^)	434.99 ± 118.39	417.46 ± 89.67	0.001
CD (cells/mm^2^)	2423.82 ± 494.32	2494.71 ± 440.25	0.003
CV (%)	36.56 ± 20.98	35.14 ± 17.13	0.154
HEX (%)	50.59 ± 26.25	54.03 ± 25.42	0.010
BMI (kg/m^2^)	23.27 ± 2.93	22.79 ± 2.74	0.001

**Table 4:** Multivariate regression analysis of CD.

Variables	Unstandardized coefficient *B*	SE	Standardized coefficient *β*	*t*	*P*	95.0% CI of *B*
Sex	−3.358	2.973	−0.015	−1.13	0.259	−9.189~2.473
Age (yr)	−2.02	0.605	−0.047	−3.342	0.001	−3.21~−0.83
HBP	15.413	13.176	0.016	1.17	0.242	−10.43~41.26
AL (mm)	−13.839	7.313	−0.027	−1.893	0.059	−28.18~0.5
IOP (mmHg)	0.613	2.161	0.004	0.284	0.777	−3.63~4.85
CCT (*μ*m)	0.387	0.192	0.027	2.018	0.044	0.01~0.76
CA (*μ*m^2^)	−3.734	0.061	−0.848	−61.031	0.000	−3.85~−3.61
CV (%)	−0.974	0.332	−0.04	−2.937	0.003	−1.62~−0.32
HEX (%)	−0.429	0.253	−0.023	−1.695	0.09	−0.92~0.07
BMI (kg/m^2^)	−10.867	11.586	−0.016	−0.938	0.348	−33.593~11.858

**Table 5:** Multivariate regression analysis of IOP.

Variables	Unstandardized coefficient *B*	SE	Standardized coefficient *β*	*t*	*P*	95.0% CI of *B*
Sex	−0.057	0.155	−0.01	−0.369	0.712	−0.36~0.25
Age (yr)	−0.047	0.007	−0.174	−6.645	0.000	−0.06~−0.03
HBP	−0.047	0.043	−0.014	−1.079	0.281	−0.13~0.04
AL (mm)	−0.219	0.087	−0.068	−2.534	0.011	−0.39~−0.05
CCT (*μ*m)	0.015	0.002	0.167	6.693	0.000	0.01~0.02
CA (*μ*m^2^)	0.005	0.001	0.185	3.808	0.000	0~0.01
CV (%)	0.01	0.004	0.065	2.507	0.012	0~0.02
HEX (%)	0.011	0.003	0.096	3.683	0.000	0.01~0.02
CD (cells/mm^2^)	0.000	0.000	0.014	0.284	0.777	0~0
BMI (kg/m^2^)	−0.456	0.527	−0.015	−0.866	0.387	−1.49~0.58

**Table 6:** Multivariate regression analysis of CCT.

Variables	Unstandardized coefficient *B*	SE	Standardized coefficient *β*	*t*	*P*	95.0% CI of *B*
Sex	1.383	1.75	0.021	0.79	0.429	−2.05~4.82
Age (yr)	−0.117	0.081	−0.039	−1.447	0.148	−0.28~0.04
HBP	−0.077	0.075	−0.014	−1.028	0.304	−0.22~0.07
AL (mm)	−1.694	0.977	−0.048	−1.735	0.083	−3.61~0.22
CA (*μ*m^2^)	−0.016	0.015	−0.053	−1.079	0.281	−0.05~0.01
CV (%)	0.053	0.044	0.031	1.193	0.233	−0.03~0.14
HEX (%)	0.054	0.034	0.042	1.589	0.112	−0.01~0.12
CD (cells/mm^2^)	0.007	0.003	0.099	2.018	0.044	0.00~0.01
IOP (mmHg)	1.904	0.284	0.171	6.693	0.000	1.35~2.46
BMI (kg/m^2^)	−0.488	0.534	−0.016	−0.913	0.361	−1.54~0.56
